# Future developments in biosensors for field-ready Zika virus diagnostics

**DOI:** 10.1186/s13036-016-0046-z

**Published:** 2017-01-23

**Authors:** Ariana M. Nicolini, Katherine E. McCracken, Jeong-Yeol Yoon

**Affiliations:** 1 Biomedical Engineering Graduate Interdisciplinary Program and Department of Biomedical Engineering, The University of Arizona, Tucson, AZ 85721 USA; 2 Department of Agricultural and Biosystems Engineering, The University of Arizona, Tucson, AZ 85721 USA

**Keywords:** Zika, Flaviviruses, Biosensors, RT-PCR, LAMP, Immunoassays

## Abstract

Since early reports of the recent Zika virus outbreak in May 2015, much has been learned and discussed regarding Zika virus infection and transmission. However, many opportunities still remain for translating these findings into field-ready sensors and diagnostics. In this brief review, we discuss current diagnostic methods, consider the prospects of translating other flavivirus biosensors directly to Zika virus sensing, and look toward the future developments needed for high-sensitivity and high-specificity biosensors to come.

## Background

**Fig. 1:**
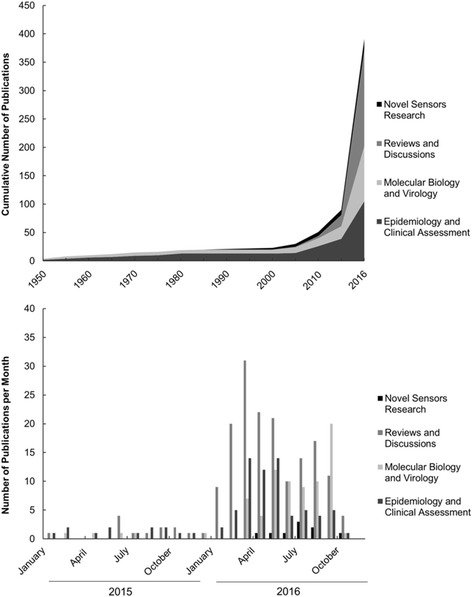
Number of peer-reviewed publications on ZIKV related to novel sensors development, topic reviews and commentaries, molecular biology and virology, and epidemiology or clinical evaluations of Zika cases (as of October 15, 2016). Cumulative publications are presented in 5 year increments until 2015 and 1 year increments between 2015 and 2016 (*top*). Publications in 2015–2016 are also presented separately by month (*bottom*)

Amidst the recent Zika epidemic, rising public health concerns have led to extensive research aimed at uncovering the underlying mechanisms of Zika virus (ZIKV) infection and transmission pathways [1–3]. According to the Pan American Health Organization (PAHO), autochthonous ZIKV cases in the Americas increased from virtually none in early 2015 to over 170,000 confirmed and 515,000 suspected cases by December 2016 [4]. This escalation has led to newly abundant clinical, epidemiological, and virological research and funding opportunities that were previously limited by the rarity of infection and limited concerns for ZIKV as an infectious agent (Fig. 1). Interestingly, research aimed at developing novel ZIKV sensors is quite limited, as seen in Fig. 1. A whole arena remains open for research, funding, and commercial opportunities.

Between its first isolation as a zoonotic pathogen in Uganda (1947) and the first major human outbreak of Zika on Yap Island in Micronesia (2007), ZIKV has been primarily observed in Africa and the Pacific [5–7]. Generally, the flu-like symptoms of infection are mild and include low to moderate fever, headache, joint pain, rash, and fatigue [6–10]. However, the recent breadth of epidemiological data stemming from the many thousands of cases across South America, the Caribbean, and Central and North America, has uncovered new insights into rare and severe effects on specific subsets of the population. These include a low risk of Guillain-Barré syndrome in adults, and critical risks for pregnant women, including stillbirth, restricted intrauterine fetal growth, and microcephaly [7, 10–14].

As a member of the *Flavivirus* genus, ZIKV shares many common genetic sequences and protein structures with other high-interest flaviviruses, including Dengue virus (DENV), West Nile virus (WNV), yellow fever virus (YFV), and Spondweni virus, its most similar relative [15, 16]. On a molecular level, ZIKV features a 10.7 kb single-stranded and positive-sense RNA genome. The polyprotein that this genome encodes cleaves to form several structural proteins, including the envelope (E) and membrane (M) proteins, and nonstructural (NS1 and NS5) proteins [17]. These proteins are the common focus in immunosensing and molecular research for other flaviviruses [12, 13, 16–18]. Thus, despite the historically limited attention given to ZIKV in the research community, previous work with other flaviviruses may help to inform a rapid turnaround in future ZIKV sensing technologies [7, 8, 15].

In the shadow of the recent epidemic, our understanding of ZIKV pathogenicity has expanded at both the population level and the molecular level. Although several benchtop methods for ZIKV detection have been employed for emergency use, there is still a need for the development and funding of alternative field-ready diagnostic tools. Prompt identification of ZIKV infection at the site of exposure from patient-direct samples is critical in minimizing the global spread of the virus. During the ongoing development and rapid expansion of the ZIKV sensor market, target specificity and sensitivity amidst complex sample matrices are key. In this brief review, we highlight current techniques, emergent diagnostic methods, and considerations for developing future field-ready biosensors.

## Gold standards of ZIKV detection

**Fig. 2:**
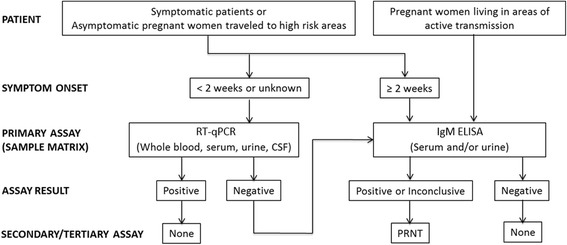
Flow diagram of gold standard ZIKV detection by patient type and time from onset of symptoms. RT-qPCR detection is typically used in the first 2 weeks of illness and IgM ELISA after the first 2 weeks or when RT-qPCR is negative. PRNT assay should be used as a final test if an ELISA assay returns positive or inconclusive

The recent increase in the number of ZIKV cases, particularly in the U.S., has led the U.S. Food and Drug Administration (FDA) to issue an Emergency Use Authorization (EUA) for several previously non-cleared or unapproved diagnostic assays. The FDA and U.S. Centers for Disease Control and Prevention (CDC) have recommended that ZIKV detection in human patients be performed by reverse transcription quantitative real-time polymerase chain reaction (RT-qPCR), or by serological tests using an IgM antibody capture enzyme-linked immunosorbent assay (MAC-ELISA) or a plaque-reduction neutralization test (PRNT) (Fig. 2) [19].

**Fig. 3:**
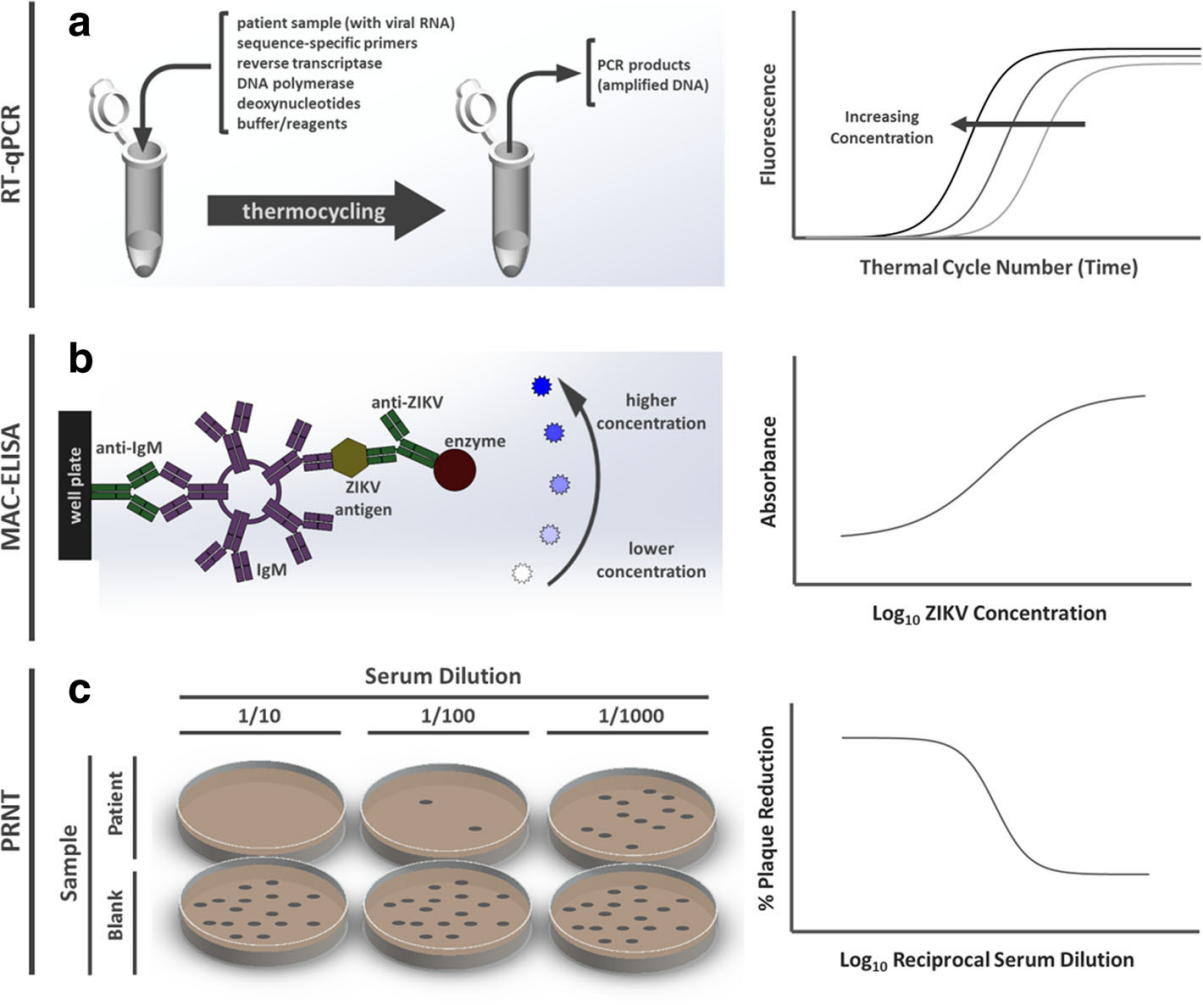
Assay schematics for ZIKV diagnostics by reverse transcription quantitative real-time polymerase chain reaction (RT-qPCR), IgM antibody capture enzyme-linked immunosorbent assay (MAC-ELISA), and a plaque-reduction neutralization test (PRNT). **a** In one-step RT-qPCR, a patient sample is thermally cycled in a buffered reagent solution containing ZIKV primers, and the amplified target is identified by fluorescence, typically after 40 cycles. **b** In MAC-ELISA, human IgM developed in response to ZIKV infection are captured and quantified though antibody interactions and enzymatic conversion of a chromogenic substrate. **c** In PRNT, patient serum dilutions are mixed with live virus samples and are applied to confluent host cells. Antibodies in infected patients neutralize the virus, leading to a reduction in observable plaques

For asymptomatic pregnant women who have traveled to high-risk areas for ZIKV and for symptomatic individuals within the first 2 weeks of symptom onset, the preferred detection method authorized by the FDA EUA is the Trioplex RT-qPCR assay, which is specific to DENV, Chikungunya virus (CHIKV), and ZIKV. In RT-qPCR, a patient sample is added to a buffered reagent solution containing target primers, reverse transcriptase (to generate cDNA from viral RNA), DNA polymerase (to amplify this cDNA), deoxynucleotides (dNTPs), and an intercalating fluorescent dye or fluorescent reporter (Fig. 3a). The amplified target is then quantified by absolute or relative fluorescence following a given number of thermocycles, typically lasting 90–120 min. This assay can be performed in the presence of several sample matrices including serum, whole blood, cerebrospinal fluid, urine, and amniotic fluid [20]. Although RT-PCR is inherently very sensitive, the possibility of false-negatives is high. Therefore, testing of symptomatic patients with negative RT-PCR results should be confirmed with alternative forms of identification.

For later stages of ZIKV infection, antibody-based methods can be used. Typically, neutralizing antibodies to ZIKV develop in the human body within the first week of symptoms and continue to remain at detectable levels for up to 12 weeks. During this timeframe, serological assays can be performed to detect the patient’s anti-ZIKV IgM antibodies. However, due to the epidemiological and molecular similarities of ZIKV to other flaviviruses, IgM ELISA assays should be conducted for the antibodies formed against ZIKV, DENV, and CHIKV. In MAC-ELISA, a patient’s sample is added to a well plate pre-coated with antibodies to capture human IgM (Fig. 3b). A virus-specific antigen is then added and washed away, binding specifically to the IgM of infected patients. Finally, an antibody specific to this same viral target that is tagged with an enzyme (e.g. horseradish peroxidase) is added and a chromogenic substrate is used for quantification. Samples from infected patients will thus elicit an optically detectible signal (e.g. absorbance, fluorescence) that may be correlated to IgM concentration. However, the risk of false-positives is high for IgM and IgG assays. If ELISA testing is inconclusive or positive, PRNT should be performed to confirm the presence of ZIKV, specifically [18].

PRNT typically serves as a secondary test to IgM ELISA and measures the ability of a patient’s antibodies to neutralize a specific virus. In PRNT, serial dilutions of a patient’s serum are added to samples of a viral suspension, and each mixture is applied to a confluent host cell culture (e.g. Vero cells) (Fig. 3c). Following incubation, plaque forming units (PFU) are counted. If neutralizing antibodies specific to this virus are present in the patient’s serum, the associated PFU value will be reduced, and the antibody titer can be determined from the serial dilutions. This method provides better sensitivity and specificity over IgM ELISA, but requires extended time (days), labor, materials, and therefore cost.

## Developmental diagnostic methods

Outside of tests that the CDC offers, there are several private companies selling RT-PCR, ELISA, and lateral flow assay kits (Table 1). Many research groups have also focused on alternative sensing modalities that reduce extensive sample preparation, the use of expensive laboratory equipment, and the risks of false-positive and false-negative results characteristic of ELISA and PCR assays. Some of these recent research findings are based on techniques previously used for the detection of other flaviviruses, whereas others are novel sensors unique to ZIKV.

**Table 1:** Laboratory-based ZIKA assay kits

Assay technique	Manufacturer	Product name
ELISA	Alpha Diagnostic International	Recombivirus Human Anti-Zika Virus (ZIKV) Envelope protein IgG/IgM ELISA kits
	CDC	^a^Trioplex Real-time RT-PCR Assay
	CTK Biotech	RecombiLISA Zika IgM ELISA kit
	DIA.PRO Diagnostic Bioprobes	ZIKV IgG/M ELISA kits
	Euroimmun	Anti-Zika Virus IgG/IgM ELISA kits
	InBios International	^a^ZIKV Detect IgM Capture ELISA kit
	MyBioSource	Zika Virus IgM (ZV-IgM) ELISA kit
	NovaTec Immundiagnostica	NovaLisa Zika Virus IgM μ-capture ELISA kit
Lateral flow assay	Chembio Diagnostic Systems	DPP Zika IgM/IgG assay
Immunofluorescence	Euroimmun	Anti-Zika Virus IIFT (IgG or IgM)
Multiplex RT-qPCR	Bioneer	AccuPower® ZIKV (DEN, CHIKV) Multiplex Real-time RT-PCR Kit
	Luminex	^a^zMAP® MultiFLEX^TM^ Zika RNA Assay
	SolGent	DiaPlexQ^TM^ ZCD Detection Kit
	ThermoFisher Scientific	TaqMan Arbovirus Triplex Assay (ZIKV/DENV/CHIKV)
RT-qPCR	Altona Diagnostics	^a^RealStar Zika Virus RT-PCR Kit 1.0
	BioinGentech	HumqPCR-realtime^TM^ Zika Detection
	CDC	Trioplex Real-time RT-PCR Assay
	Coyote Bioscience	One Step qPCR Detection kit for the Zika Virus
	DaAn Gene	Detection Kit for Zika Virus RNA
	Focus Diagnostics	^a^Zika Virus Qualitative Real-Time RT-PCR
	Liferiver	Zika Virus Real Time RT-PCR
	MyBioSource	Zika, PCR Kit
	Roche Molecular Systems	^a^LightMix® Zika rRT-PCR Test
	Siemens Healthcare Diagnostics	^a^VERSANT® Zika RNA 1.0. Assay (kPCR)
	ThermoFisher Scientific	TaqMan Zika Virus Singleplex Assay
	US Biological Life Sciences	Genesig Teal-Time PCR Kit for Zika Virus, Easy
	Viracor-IBT Laboratories	^a^Zika Virus Real-time RT-PCR Test
	WELLS BIO	careGENE^TM^ Zika Virus RT-PCR Kit
RT-PCR	ARUP	^a^Zika Virus Detection by RT-PCR Test
	Vela Diagnostics USA	^a^ *Sentosa®* SA ZIKV RT-PCR Test
Transcription-mediated amplification	Hologic	^a^Aptima® Zika Virus Assay

^a^Indicates assays approved under the FDA Emergency Use Authorization (as of October 21, 2016)

### Molecular detection of ZIKV nucleic acid

Reverse transcription PCR (RT-PCR) has become the gold standard for molecular amplification and detection of viruses because of its high selectivity and relatively high sensitivity. Prompted by the 2007 ZIKV outbreak in Yap State, Micronesia, several RT-PCR methods have been developed to specifically identify a multitude of ZIKV strains independent of other flaviviruses. Published ZIKV-specific primer sets target highly conserved regions of the structural membrane (M) and/or envelope (E) [21] proteins, partial envelope (pE) [9] protein, or the non-structural (NS1 and NS5) proteins [18, 22–25]. Detection of ZIKV using RT-PCR has also been shown to work in the presence of many sample matrices including plasma [26], serum [21, 27], saliva [28], urine [27], conjunctival fluid, and semen [29], thus reducing the need for sample purification or extraction.

Many commercial nucleic acid amplification tests (NAATs) have been developed for ZIKV detection within the past year (Table 1). Between February 26, 2016 and October 21, 2016, the FDA approved ten molecular diagnostic tests for clinical identification of ZIKV under the EUA [30]. Eight of these assays utilize traditional RT-PCR or RT-qPCR amplification (conventional and quantitative real-time thermal cycling) and detection (gel electrophoresis or intercalating dye fluorescence intensity) methods.

The following two non-traditional FDA EUA-approved NAATs claim improved sensitivity, specificity, usability, and speed. The xMAP® MultiFLEX^TM^ assay (Luminex Corp.), uses a proprietary device to complete a series of steps, which include RT-PCR, followed by amplicon-particle hybridization, and final detection via an indicator molecule [31]. The other, Aptima Zika virus assay (Hologic, Inc.), also uses a proprietary device; however, this assay is fully automated and can perform transcription-mediated amplification (exact technique not specified), and qualitative viral detection in the presence of human serum, plasma, or urine, similar to the xMAP® MultiFLEX™ assay [32]. Despite claiming ease-of-use and rapid sample-to-answer times, both methods require approximately 3.5 h and expensive laboratory equipment, and thus laboratory space.

**Fig. 4:**
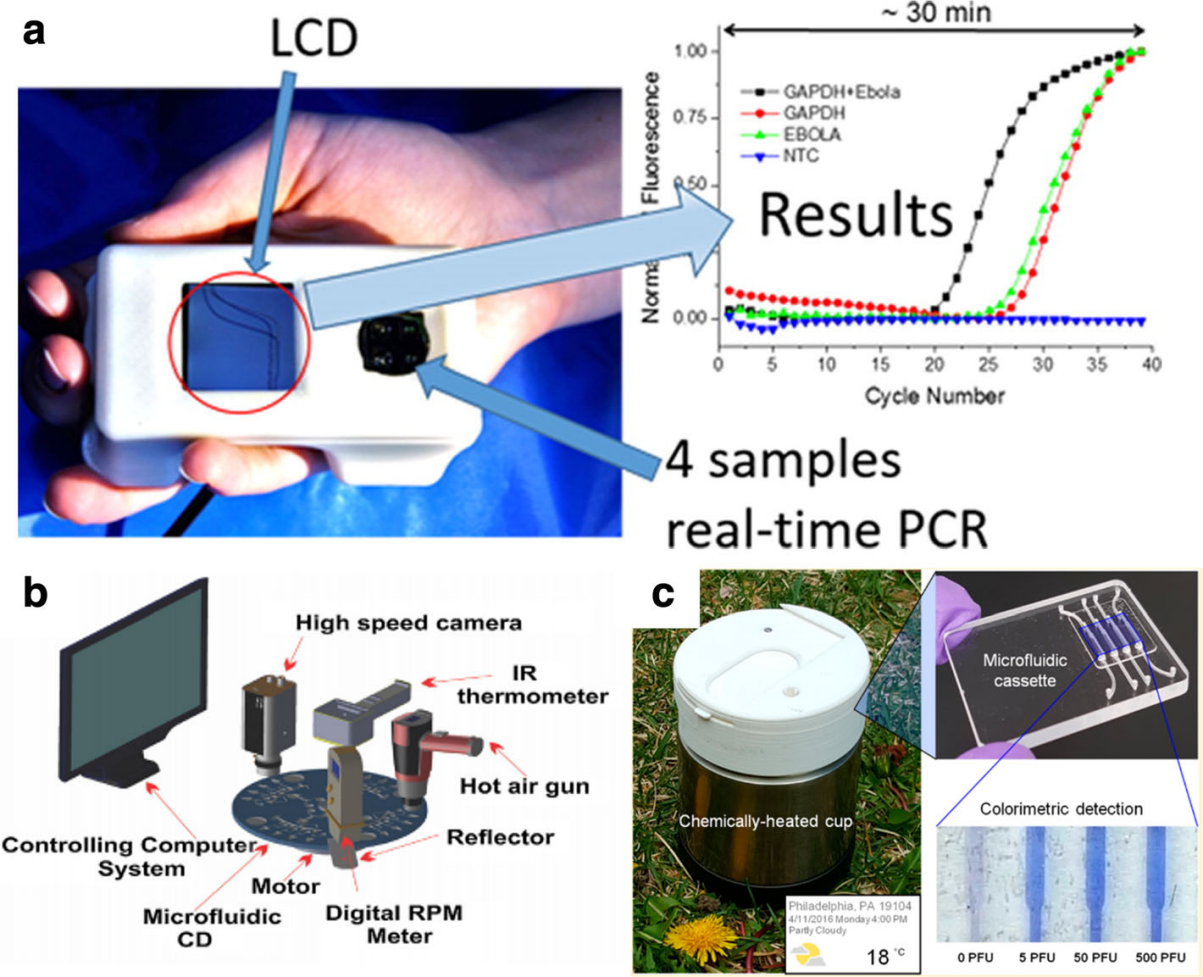
**a** Palm-sized device for point-of-care Ebola detection using RT-PCR and fluorescence detection (reproduced from ref. 33 with permission from American Chemical Society). **b** Lab-on-a-CD integrated LAMP for foodborne pathogen detection (reproduced from ref. 45 with permission from Elsevier). **c** Instrument-free RT-LAMP assay and self-contained cassette for point-of-care ZIKV assay (reproduced from ref. 40 with permission from American Chemical Society)

In the case of epidemic diseases, extremely rapid and low-cost screening of clinical samples in-field is necessary, making these EUA techniques inadequate. In light of this need, many research groups have focused on making PCR assays field-deployable and/or field-ready [33–37]. Although some have succeeded in creating full sample-to-answer devices (Fig. 4a), PCR platforms are still limited by their need for multi-temperature sample heating for denaturation, annealing and extension. Fortunately, over the past 30 years isothermal amplification techniques with typical amplification times of less than 1 h have been thoroughly described for a variety of DNA and RNA targets. Popular forms of isothermal NAATs include nucleic acid sequence-based amplification (NASBA), loop-mediated isothermal amplification (LAMP), strand invasion based amplification (SIBA), strand displacement amplification (SDA), helicase-dependent amplification (HAD), recombinase polymerase amplification (RPA) and others [38].

Since the first publication by Pardee et al. in May 2016, four groups have published research on isothermal-NAAT ZIKV detection using NASBA [39], RT-LAMP [40, 41] and RT-SIBA [42] (Table 2), several of which are still laboratory-based. All four groups also used different amplicon detection modalities, including toehold switch sensors, colorimetric detection, AC susceptometry, and gel electrophoresis. The RT-LAMP assay developed by Song et al. is particularly noteworthy due to its self-contained and field-ready design, which allows identification of ZIKV in under an hour on a portable cassette for less than $2 per assay (Fig. 4c) [40].

**Table 2:** ZIKV biosensors developed in 2016

Assay technique	Detection mode	Target	Sample matrix	Range of detection	Limit of detection	Sample volume	Assay time	Cost per assay	Ref.
Immunoassay	Impedimetry	NS1 (flaviviruses)	PBS Serum	10-2000 ng/mL 10-1000 ng/mL	3 ng/mL 30 ng/mL	40 μL	30 min	unspecified	[61]
	Capacitance	NS1 (flaviviruses)	PBS Serum	5-1000 ng/mL 5-1000 ng/mL	0.2 ng/mL 0.5 ng/mL				
Immunoassay	Chemiluminescence	E protein (ZIKV)	PBS Urine, plasma	10-10^5^ PFU/mL 10-10^4^ PFU/mL	10 PFU/mL 10 PFU/mL	100 μL	~2 h	unspecified	[68]
LAMP	AC susceptometry	NS5 oligonucleotide (ZIKV)	Unspecified Serum	1-10^3^ aM 1-10^4^ aM	1 aM 1 aM	40 μL	<30 min	unspecified	[41]
NASBA-CRISPR	Colorimetry	RNA (ZIKV)	Serum	3 fM - 30 pM	1 fM	300 μL	~3 h	$0.10-$1	[39]
RT-LAMP	Colorimetry	E protein RNA (ZIKV)	Saliva	50-5 × 10^4^ PFU/mL	50-100 PFU/mL	65 μL	40 min	~$2	[40]
RT-SIBA	Fluorescence	RNA (ZIKV)	Lysis buffer	5 × 10^3^-5 × 10^6^ copies/mL	5000 copies/mL	2 μL	<30 min	unspecified	[42]

Of the molecular diagnostic techniques, isothermal genomic amplification has arguably become the most promising method for in-field pathogen identification due to its enhanced specificity, decreased limit-of-detection, reduced assay time, ease of amplification, and number of end-product detection methods. Although not all for ZIKV, several groups have already developed low-cost devices using inexpensive insulating materials (e.g. thermoses) [40, 43, 44] and simple heat-producing elements [45–48], including non-electrical exothermal reactions [43, 49, 50] (Fig. 4b,c). Many real-time nucleic acid quantification methods have also been used, though again not all for ZIKV, and include measurement of fluorescence [51], Mg^+^ pyrophosphate [52], electrochemical [53], or colorimetric signal changes, detectable by the human eye [54, 55] or optical sensors [56–58].

### Antibody-based assays

Despite advances in molecular diagnostics, the cost of reagents and equipment and the likelihood of false-negative results present inherent challenges. For these reasons, serological assays remain important alternatives or supplements for detection, particularly when focusing on field-ready assays [18]. These techniques have been most commonly used to detect a patient’s antibody response within a diseased state, as previously described, but can also be extended to direct assays for ZIKV antigens in any sample matrix (i.e. immunoassay), including mosquito pool samples. Flavivirus immunoassays, including ELISA and antibody-based lateral flow assays, have primarily been developed through the antibodies to NS1, NS5, or E proteins [59]. These are also the principal routes of detection in commercial ZIKV MAC-ELISA kits authorized by the FDA (Table 1) [60].

Depending on the extent of conservation for a targeted epitope among all flaviviruses, some existing assays for DENV or YFV may be adapted directly to ZIKV, but will only have the resolution to broadly identify ZIKV as a flavivirus. Recent immunosensors developed and tested by Cecchetto et al., for example, use impedimetric and capacitive sensing of the NS1 protein from DENV, and have the potential for near immediate conversion to ZIKV detection due to potential cross-reactivity of the anti-NS1 IgG1 antibodies employed [61] (Table 2). Similarly, flavivirus biosensors have been developed using lab-on-a-chip and lab-on-a-CD technology for label-free optical and electrochemical sensing of DENV through serological IgM or NS1 protein binding [62, 63].

Because ZIKV and other flaviviruses are similarly transmitted to humans by mosquitoes of the *Aedes* genus, though, the origin of a biomarker detected by a nonspecific immunoassay may be unclear [64]. This potential for cross-reactivity is a primary concern for ZIKV immunosensing and begs further research into high-affinity antibodies with greater species specificity.

In response to these concerns, new research by Dai et al. has focused on discerning the operation of flavivirus antibody recognition for ZIKV through improved characterization of surface protein structures at the angstrom level [65]. Their work has discerned one mode of antibody binding specifically to the ZIKV E protein along a conserved fusion loop, which may be a focal point for future targeted sensors. An extensive survey of E protein structures across 50 ZIKV strains by Badawi et al. has also confirmed multiple conserved epitopes between these, and work by Zhao et al. has revealed several candidate mouse antibodies that demonstrate favorable specificity for ZIKV detection through binding localization at the DIII feature of the ZIKV E protein [59, 66]. However, other proteins may also be ideal candidates for sensing methods. For example, Meltzer et al. have recently highlighted the merits of developing IgM and IgG that are specific to the ZIKV NS1 protein, through which detection may also be more species-specific [67].

**Fig. 5:**
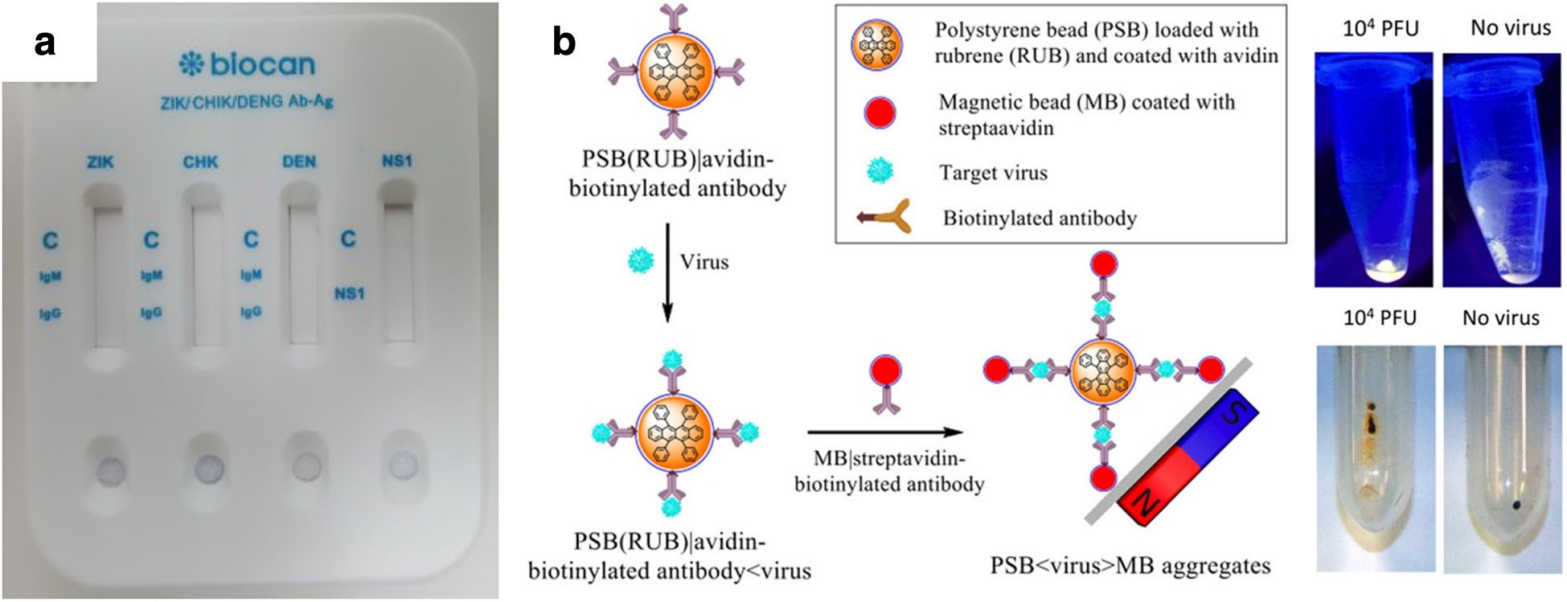
**a** Biocan diagnostic’s Tell Me Fast™ Zika/Dengue/Chikugunya virus IgG/IgM lateral flow assay (reproduced from www.zikatest.com with permission from Biocan Diagnostics, Inc.). **b** Chemiluminescent particle immunoassay for ZIKV detection by magnetic separation and ultraviolet fluorescence (reproduced from ref. 68 with permission from the authors)

Following these efforts, early steps toward instrument-free and point-of-care (i.e. field-ready) ZIKV-specific immunosensors have been reported, although these have been few in number. For example, Acharya et al. developed a chemiluminescent immunoassay that specifically detects ZIKV by recognition of the E protein and quantification following magnetic particle separation and immunoblotting (Fig. 5) [68]. Exploration into immunosensing methods adaptable to field-ready diagnostics promises substantial improvements in future ZIKV detection and treatment, especially if cross-reactivity can be eliminated with new high-affinity, high-specificity antibodies.

## Conclusions

Considerable research is still required to reach our goals of ZIKV sensing across an array of sample matrices with field-ready assay platforms. Fortunately, much has been learned over the past year about ZIKV on a molecular level, and thus many new opportunities have emerged for applying this knowledge toward treatment and diagnostics. Molecular identification of not only ZIKV, but also of other flaviviruses, hinges on the implementation of alternative techniques for amplicon production and detection. Strides have been made in the design of suitable primer sets specific to flaviviruses and ZIKV in particular; however, more development is needed for rapid, in-field detection. For antibody-based assay development, researchers may build their methods from existing cross-reactive assays, but adoption of forthcoming ZIKV-specific antibodies will be necessary for improved specificity. Above all, sensors that can be quickly and inexpensively assembled, screened for quality, and deployed will make the greatest impact in helping to understand and prevent the spread of ZIKV.

## Abbreviations

CDC: U.S. Centers for Disease Control and Prevention
CHIKV: Chikungunya virus
DENV: Dengue virus
EUA: Emergency Use Authorization
FDA: U.S. Food and Drug Administration
HAD: Helicase-dependent amplification
LAMP: Loop-mediated isothermal amplification
MAC-ELISA: IgM antibody capture enzyme-linked immunosorbent assay
NAATs: Nucleic acid amplification tests
NASBA: Nucleic acid sequence based amplification
PRNT: Plaque-reduction neutralization testing
RPA: Recombinase polymerase amplification
RT-PCR: Reverse transcription polymerase chain reaction
RT-qPCR: Reverse transcription quantitative real-time polymerase chain reaction
SDA: Strand displacement amplification
SIBA: Reverse transcription-strand invasion based amplification
WNV: West Nile virus
YFV: Yellow fever virus
ZIKV: Zika virus
